# Pelvic Health Assessment in Adult Females Following Pediatric Appendicitis: A Monocentric Retrospective Case—Control Study

**DOI:** 10.3390/children9030346

**Published:** 2022-03-03

**Authors:** Giovanni Parente, Marco Di Mitri, Simone D’Antonio, Sara Cravano, Eduje Thomas, Marzia Vastano, Robert Lunca, Tommaso Gargano, Michele Libri, Mario Lima

**Affiliations:** Pediatric Surgery Department, IRCCS Sant’Orsola Polyclinic, Alma Mater Studiorum—University of Bologna, 40138 Bologna, Italy; marcodimitri14@gmail.com (M.D.M.); alcmeone1@libero.it (S.D.); sara-cravano@libero.it (S.C.); edu.thomas92@gmail.com (E.T.); marzia.vastano@icloud.com (M.V.); robert.lunca@gmail.com (R.L.); tommaso.gargano2@unibo.it (T.G.); mlibri31@yahoo.it (M.L.); mario.lima@unibo.it (M.L.)

**Keywords:** acute appendicitis, peritonitis, gynecological health, chronic pelvic pain, transitional care

## Abstract

Background: The anatomical location of the appendix in females determines its close contact with the internal genitalia, involving the latter in case of acute appendicitis (AA). The aim of this study was to evaluate the incidence of pelvic health impairment in adult women who underwent appendicectomy during childhood. Materials and Methods: A retrospective observational study was conducted including all female patients who underwent appendicectomy for acute appendicitis at our Center between January 1985 and December 1995. The patients were divided into two groups, i.e., complicated AA (Group A) and not complicated AA (Group B), and were asked to respond to a questionnaire investigating their general health status, fertility impairment, ectopic pregnancies, miscarriages, endometriosis, and chronic pelvic pain. The same questionnaire was administered to female volunteers with past medical history (PMH) negative for AA. The data were compared using chi-square test and Fisher exact test (a *p* value < 0.05 was considered for statistical significance). Results: In total, 75 patients operated for AA during childhood (22 in Group A and 53 in group B) and 44 female volunteers with PMH negative for AA (group C) were enrolled in the study. Seventeen patients (77.3%) in group A, 40 (75.4%) in group B, and 29 (65.9%) in group C (*p* > 0.05) had pregnancies. The number of miscarriages among women who became pregnant in their life was 5 in group A, 13 in group B, and 12 in group C (*p* > 0.05). Chronic pelvic pain was reported by 7 out of 22 (31.8%) patients in group A, 7 out of 53 (13.2%) in group B, and 5 out of 44 (11.4%) in group C (A vs. C: *p* = 0.04, OR = 3.64; A vs. B: *p* = 0.06 and B vs. C: *p* = 0.52). Conclusions: In our series, AA, complicated or not, neither determined repercussions on fertility, risk of miscarriages, and ectopic pregnancies nor increased the risk of developing endometriosis. However, women who experienced complicated AA showed a higher prevalence of chronic pelvic pain onset in adulthood compared to healthy women.

## 1. Introduction

Acute appendicitis (AA) is the most common cause of abdominal pain in children, with an estimated incidence ranging from 1 to 6/10,000 in the age group from 0 to 4 years of age and from 19 to 28/10,000 in the age group between 5 and 14 years of age. Boys are more frequently affected than girls [1].

Appendicitis presents in a spectrum of anatomopathological entities, varying from a simple inflammation to perforation with systemic involvement (peritonitis, sepsis).

The most frequent etiology is thought to be the obstruction of the appendiceal lumen by appendicoliths; other causes include lymphoid hyperplasia, foreign body, parasites, or malignancy [2]. The obstruction of the lumen leads to the distension of the appendix, which continues to secrete mucus promoting bacterial proliferation, and the impairment of the lymphatic and venous drainage, consequently limiting also the arterial supply. As the disease progresses, the appendiceal tissue undergoes necrosis and then perforation [3,4].

Laparoscopic appendectomy has become the gold standard among treatments, replacing the conventional open technique [5].

The widespread adoption of the laparoscopic approach and the growing expertise in it have led to a substantial decrease in intraoperative complications, ensuring considerable benefits such reduced post-operative pain and shorter hospitalization [5,6]; the morbidity and mortality of patients who undergo appendectomy largely depends on the severity of AA [7].

The possible extension of the inflammatory process from the appendix to nearby structures, especially in case of appendicular peritonitis, can affect, in female patients, the internal genitalia, with possible sequelae on gynecological health [8].

Cases of reduction in ovarian follicular growth, hypomotility of the tubes, and adhesions close to the right ovary and tube after AA have been described in the literature [9].

In this study, we investigated potential correlations between acute appendicitis and pelvic health impairment.

## 2. Materials and Methods

A retrospective observational study was conducted in our Department of Pediatric Surgery, IRCCS Sant’Orsola, University Hospital of Bologna, following Ethical Committee approval (CHPED-21-04-GYN).

Clinical records were retrospectively analyzed to identify all female patients operated for AA in our center between January 1985 and December 1995. The time interval was chosen to ensure that all patients were currently at least 18 years old.

All women between 18 and 50 years old with a negative medical history for abdominal surgery other than appendicectomy and caesarian section were enrolled.

Data such as age at surgery, histological classification of the appendicitis (catarrhal, phlegmonous, gangrenous, perforated), presence of peritonitis, and abdominal/pelvic abscesses were recorded.

**Figure 1:**
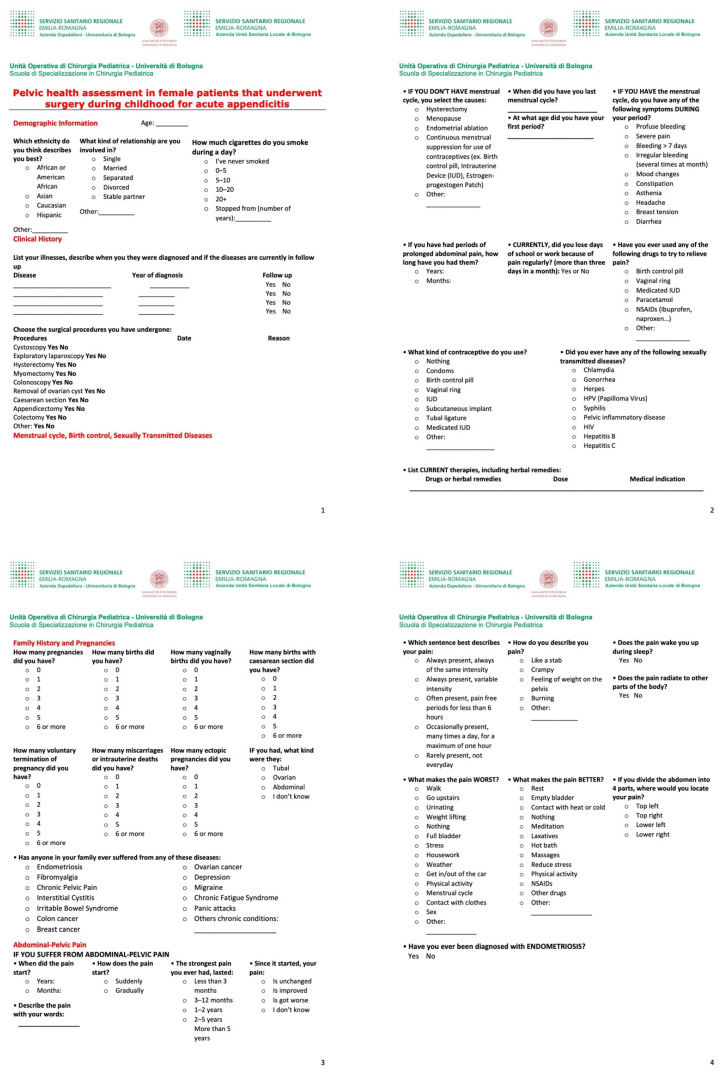
Questionnaire administered to the patients: detailed ob-gyn anamnestic form.

A questionnaire (standard detailed ob-gyn medical history form) was administered to all patients included in the study, investigating demographic data (age, ethnicity, smoking habits, type of relationship currently involved in), family history, past medical and surgical history, use of contraceptives, Sexually Transmitted Disease (STD), menstrual cycle (presence and causes of amenorrhea, menarche, date of the last cycle, symptoms during the cycle), pregnancies (number, full-term deliveries, vaginal deliveries, caesarean sections, voluntary interruption of pregnancies, miscarriages, extrauterine pregnancies), presence, and eventually description, of chronic abdominopelvic pain and diagnosis of endometriosis (Figure 1).

The patients were divided into two groups: Group A included patients with complicated AA (i.e., gangrenous, perforated with/without abdominal/pelvic abscess or peritonitis), and Group B included patients with non-complicated AA (i.e., catarrhal or phlegmonous).

We randomly administered the same questionnaire to 18–50-year-old female volunteers working in our institute with a negative medical history for AA and abdominal surgery except for caesarian section (control group, Group C).

Continuous data are presented as mean ± standard deviations.

Statistical analysis was performed using Fisher exact test, Chi-square test, and *t* test; a *p* value < 0.05 was considered statistically significant.

## 3. Results

Between January 1985 and December 1995, 256 female patients underwent appendicectomy at our Pediatric Surgery Department. Among these, 138 were tracked down, and 76 of them accepted to participate to the study. In total, 75 out of the 76 patients enrolled satisfied the inclusion criteria and answered the questionnaire.

All patients underwent open appendicectomy, and the histology exams reported 22 patients with catarrhal AA (29.3%), 31 with phlegmonous AA (41.4%), and 22 with gangrenous or perforated AA with appendicular peritonitis or abscess (29.3%).

The patients were divided into two groups:

Group A included 22 patients (29.3%) treated for complicated AA (gangrenous or perforated and appendicular peritonitis or abscess);

Group B included 53 patients (70.7%) treated for uncomplicated AA (catarrhal or phlegmonous AA).

A third control group, named Group C, included 44 healthy female volunteers who had never undergone surgery, except for caesarean section.

The mean age at surgery of Group A and B was, respectively, 7.5 ± 4.5 (range: 2.5–20.9) and 9.5 ± 2.4 (range: 4.7–4.2) years, with a statistically significant difference (*p* < 0.01). The mean age of Group A, B, and C when answering the questionnaire was, respectively, 38.7 ± 4.7 (range: 27–46), 40.7 ± 4.2 (range: 32–48), and 39.7 ± 6.9 (range: 28–50) years (*p*-values are reported in Table 1).

According to the kind of relationship in which the patients were involved, Group A included 3 (13.6%) single women, 9 (40.9%) married women, 2 (9.1%) separated women, and 8 (36.4%) women with a regular partner; Group B included 2 (3.8%) single women, 27 (50.9%) married women, 4 (7.6%) separated women, and 20 (37.7%) women with a regular partner; Group C included 8 (18.2%) single women, 25 (56.8%) married women, 2 (4.6%) separated women, and 9 (20.5%) women with a regular partner.

To study patients’ fertility, we considered only women in a stable relationship, i.e., just married and with a single regular partner; it was therefore possible to identify 17 (77.3%) women in a stable relationship in group A, 47 (88.7%) in group B, and 33 (75%) in group C.

Women who had pregnancies were 17 (77.3%) in group A, 40 (75.4%) in group B, and 29 (65.9%) in Group C. Considering only women in a stable relationship, the number of women who had pregnancies was 14 (82.4%) in group A, 35 (79.5%) in group B, and 26 (78.8%) in group C.

The total number of pregnancies was 29 in group A, 23 (79.3%), of which those carried to term included 17 (73.9%) with vaginal delivery and 6 (26.1%) with caesarean delivery. Group A did not declare any voluntary interruption of pregnancy (VIP) nor any ectopic pregnancy, but six miscarriages (20.7%) were recorded.

Group B reported a total of 82 pregnancies, 59 (72.0%) of which were carried to term, while 3 were still in progress when the questionnaire was administered and, thus, were excluded. Forty-five out of the 59 (76.3%) pregnant women delivered vaginally, and 14 out of 59 (23.7%) underwent caesarian section. Moreover, Group B declared 4 VIPs (4.9%), 13 miscarriages (15.9%), and 3 ectopic pregnancies (3.7%, all occurred in a Fallopian tube).

Group C reported a total of 72 pregnancies (one excluded because still in progress during the questionnaire administration), of which 52 (72.2%) carried to term, 38 (73.1%) with vaginal delivery, and 14 (26.9%) with caesarian delivery. Group C declared 17 miscarriages (23.6%) and 2 ectopic pregnancies (2.8%, one occurred in the Fallopian tube, while the exact location of the other was not known by the patient).

Comparing pregnancies distribution, no statistically significant differences were found between the three groups (*p* > 0.05).

When considering only married women and those with a stable partner, differences in pregnancies’ distribution between the three groups were not statistically significant (*p* > 0.05).

The comparison of the number of miscarriages between the three groups did not show a statistically significant difference (*p* > 0.05).

Excluding nulliparous women, the number of ectopic pregnancies in the three groups was 0 in group A, 1 in group B, and 2 in group C. The differences were not statistically significant (*p* > 0.05).

Chronic pelvic pain was reported by 7 out of 22 (31.8%) patients in group A, 7 out of 53 (13.2%) in group B, and 5 out of 44 (11.4%) in group C. The analysis suggested that the patients in group A were more affected by chronic pelvic pain than those in the control group (*p* = 0.04), with an OR = 3.64, while no statistical significance was found in the comparison between groups A and B (*p* = 0.062) and between groups B and C (*p* = 0.516).

Endometriosis was diagnosed in one case (4.6%) in group A, in five cases (10.6%) in group B, and in one case (2.3%) in group C. The differences between the groups were not statistically significant (*p* > 0.05).

**Figure 2:**
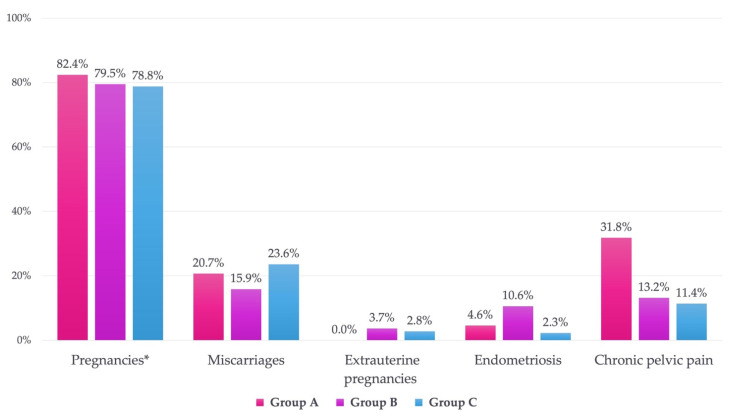
Incidence of pregnancies, miscarriages, ectopic pregnancies, endometriosis, and chronic pelvic pain in the three groups. Group A: complicated AA, Group B: not-complicated AA, Group C: control group; *: pregnancies in women in a stable relationship.

Data are summarized in Figure 2 and Table 1.

## 4. Discussion

The gold standard among treatments for AA in children is appendectomy [10].

Adhesion formations, due to the inflammatory nature of the disease and/or secondary to surgery, especially if performed with open techniques, are extremely common, representing a considerable cause of morbidity [11].

The possible extension of the inflammation from the appendix to close structures can affect, in female patients, the internal genitalia, with possible sequelae on gynecological health; this is even more common in cases of generalized inflammation such as appendicular peritonitis.

In the literature, there are several studies assessing possible correlations between female infertility and acute appendicitis [12].

Wiig et al., in a retrospective study, analyzed a cohort of patients operated during childhood for perforated AA and a second cohort of patients with Douglas abscess, comparing them with a healthy control group; they showed that, respectively, 19%, 31%, and 12% of women in the above-mentioned groups could not have children, concluding that infertility could be correlated to the severity of appendix inflammation [13].

Geerdsen et al. investigated the incidence of infertility, defined as failure in getting pregnant after 12 months of unprotected sexual intercourses, in female patients who underwent appendectomy during childhood for non-complicated AA in comparison with patients who were operated for complicated AA; no significant differences between these two groups were found. Nevertheless, if compared with healthy women of the same age, women operated for complicated AA presented higher rates of infertility. According to Geerdsen et al., such findings suggest the need for early treatment of AA in young girls to avoid long-term complications affecting their fertility [14].

Thompson et al. reviewed data of girls under 20 years treated for perforated AA at the Mayo Clinic between January 1940 and December 1949. They stated that infertility could be a potential sequela of peritonitis, concluding that, in cases of doubtful diagnosis, appendectomy should be performed anyway [15].

Liakakos et al. analyzed the most common etiological factors of infertility and its pathophysiology in case of peritonitis and peritoneal adhesions’ formation. They asserted that inflammatory adhesions could affect gynecological health by causing a reduction in ovarian follicular growth and a hypomotility of the tubes. This results in a slower passage of the oocyte and in difficulties for the embryo to reach the uterus, increasing the risk of ectopic pregnancies [9].

Elraiyah et al. conducted a meta-analysis considering randomized trials and observational studies enrolling patients who underwent open or laparoscopic appendectomy to ascertain any possible relationship between acute appendicitis and tubal infertility or ectopic pregnancy. The results indicated a statistically significant increase in the incidence of extra-uterine pregnancies in women who underwent appendectomy (OR = 1.78, *p* < 0.0001), without finding a statistically significant difference in the rate of infertility (OR 1.03, *p* = 0.91) [16].

Lalos et al. compared data obtained from 120 women with tubal infertility due to tubal occlusion and/or peritoneal–adnexal adhesions and 126 pregnant women: they showed that previous pelvic surgery and inflammation were the most important risk factors for tubal infertility (*p* < 0.001). Moreover, abdominal surgery seemed to be a risk factor in the onset of abdominal pain of unknown etiology (*p* < 0.001) [17].

Contrarily to most of the literature discussed above, our study did not reveal any significant fertility impairment in women with complicated AA, uncomplicated AA, and in the control group.

Moreover, we did not find any significant differences in the incidence of either ectopic pregnancies or endometriosis and miscarriage between the three groups.

An important finding derived from our study is the statistically significant increase in the incidence of chronic pelvic pain (CPP) in women with complicated AA compared to the control group (OR = 3.64, 95% confidence interval = 1.00–13.26, *p* = 0.047).

Only Lalos et al. reported the same result in their series [17].

This result suggests that the onset of CPP in adulthood could be a long-term complication of complicated AA. 

In the literature, CPP is considered a multifactorial illness secondary to a series of bladder, urethral, gynecological, anorectal, neurogenic, vascular, or cutaneous diseases, and it is often impossible to identify a single cause of CPP [18]. Probably, a dysfunction of the somatosensory system, due to peripheral and central sensitization, associated with neurogenic inflammation, can determine the clinical manifestation of CPP. Indeed, it is described that the onset of CPP could be triggered by a previous episode of infection and inflammation, such as peritonitis secondary to AA, that determines the hyperstimulation of the afferent pain fibers of the nervous system, favoring the long-term onset of hyperalgesia and allodynia [19].

Therefore, it remains unclear if CPP in patients operated for AA is more likely dependent on a peripheric neurologic impairment or arising from internal genitalia conditions. In our opinion, the latter cannot be excluded with certainty. 

Summarizing the studies previously discussed, along with our results, it is still debated if AA during childhood can in the future impair the gynecological function in women.

What we surely recommend, is to consider CPP as a possible long-term complication of appendiceal peritonitis in females.

Because of the higher mortality and morbidity of complicated AA compared to simple AA, we, pediatric surgeons, agree on the need for prompt diagnosis and treatment of this disease to prevent the establishment of peritonitis. The area in which our results and the contradictory evidence on this topic in the literature find the highest level of clinical applicability is the doubtful diagnosis of AA.

Indeed, on the basis of what discussed above, we suggest preferring appendectomy to clinical observation in case of doubtful AA, in order not to incur in long-term complications such as CPP.

Moreover, we suggest presenting this topic during the preoperative informed consent discussion to raise awareness in the parents about conditions that may affect the future life of their daughters.

### Limits of the Study

We are aware of the limits of the present study. First, the low number of enrolled patients decreases the statistical strength of our conclusions. The number of patients obtained from the operating registers was extremely significant. However, as expected, after 20 years it was difficult to trace some patients (change of telephone number, address, etc.). Finally, as it commonly happens when administering a questionnaire, not all candidates thoroughly answered the questionnaire, leading to a further decrease of the number of participants. Nevertheless, we believe that the size of our population is sufficient for a preliminary result that should in any case be further investigated through multicenter studies, to which we are willing to collaborate.

Second, we are conscious of the heterogeneous definition of complicated appendicitis; we adopted the most common one, which is perforation of the appendix with or without abscess formation and fecal peritonitis. We just added gangrenous appendicitis to this definition, because this condition generally determines at least an irritation of the peritoneum.

Moreover, it is difficult to study fertility in women just based on whether they have ever become pregnant and on the number of their pregnancies, and it is also extremely tricky not to run into selection biases, even if we tried to apply a standardization by considering the parameter “being in a stable relationship”. This parameter was chosen because, considering the definition of infertility (not being able to become pregnant after one year, or longer, of unprotected sex), this category of women is the one that likely practices unprotected sex (to be sure, in the questionnaire, we investigate contraception). On the other hand, if we had only enrolled patients with diagnosed infertility, followed up in the Obstetrics and Gynecology department of our institute, studying how many of them had a PMH positive for appendicular peritonitis, we would have fallen into an even worse selection bias that could have led to an overestimate of the possible findings.

Lastly, the following issues remain to be considered and better addressed by further studies.

First, is CPP derived from AA or is it a consequence of the surgical technique? A study evaluating CPP in a cohort of patients with the same characteristics as ours who were operated using the laparoscopic technique or the open surgery is needed.

Second, is it possible to identify an anatomical explanation to our findings? To answer to this question, it is necessary to perform investigations such as hysterosalpingography or sonohysterography, but we have to take into account the invasive nature of these exams. Therefore, we preferred to wait for the current preliminary results to understand if it can be worth it to further proceed with second-level diagnostic tools in future studies. 

Third, when facing an appendicular abscess, several options are suggested in the literature: emergency appendectomy and peritoneal cavity’s toilette (approach followed in our center) or antibiotic therapy and/or CT-guided drainage of pus and fluid, followed by appendectomy 8 to 12 weeks later. The latter approach is called “interval appendectomy” [20,21,22,23,24,25]. Regarding the aim of the present study, it could be interesting to collaborate with centers where interval appendectomy is routinely performed to evaluate the presence or absence of gynecological sequelae in this category of patients. In our opinion, considering that the potential damage is determined by acute inflammation that spreads from the appendix to the internal genitalia rather than by the timing of the surgical intervention, we do not expect different results from those obtained for our group A patients; however, this is just a speculation, and further studies are needed to confirm our hypothesis.

Finally, does complicated AA in adulthood predispose to the same long-term complications discussed above, or does its impact remain limited to childhood? This topic should be addressed too in future papers, and we encourage general surgeons and gynecologists to investigate it. 

Therefore, further studies, especially multicentric ones with a high number of participants, focused on the issues mentioned above are needed to better investigate and understand the topic of our work.

## 5. Conclusions

Even if data in the literature are controversial, our study did not show a significant perturbance in the gynecological function of future women operated for AA during childhood.

The only suspected long-term consequence of complicated AA is the onset of CPP in adulthood.

We are far from considering this topic exhaustively dealt with, as long as a lot needs to be explained about CPP in this population.

Our findings should promote further multicentric studies to reach more reliable conclusions. 

Furthermore, we strongly recommend performing a similar study comparing patients who underwent open and minimally invasive surgery to understand if long-term consequences should be attributed to the inflammatory nature of AA or to the surgical technique.

## Figures and Tables

**Table 1:** Data of the study and *p*-values. Group A: complicated AA, Group B: not-complicated AA, Group C: control group; *: pregnancies in women in a stable relationship.

	Group A	Group B	Group C	*p*-Values		
				A vs. C	B vs. C	A vs. B
Enrolled	22	53	44	*-*	*-*	*-*
Age at surgery	7.5 ± 4.5	9.5 ± 2.4	-	-	-	<0.01
Current age	38.7 ± 4.7	40.7 ± 4.2	39.7 ± 6.9	0.24	<0.01	0.04
Pregnancies *	14/17 (82.4%)	35/47 (79.5%)	26/33 (78.8%)	0.54	0.50	0.44
Miscarriages	6/29 (20.7%)	13/59 (15.9%)	17/72 (23.6%)	0.31	0.35	0.54
Ectopic pregnancies	0/29 (0.0%)	3/59 (3.7%)	2/72 (2.8%)	0.39	0.38	0.70
Chronic pelvic pain	7/22 (31.8%)	7/53 (13.2%)	5/44 (11.4%)	0.04	0.52	0.06
Endometriosis	1/22 (4.6%)	5/53 (10.6%)	1/44 (2.3%)	0.74	0.30	0.43

## Data Availability

The data presented in this study are available on request from the corresponding author. The data are not publicly available due to privacy restrictions.
